# A qualitative exploration of oral health care among stroke survivors living in the community

**DOI:** 10.1111/hex.13074

**Published:** 2020-06-19

**Authors:** Lucy O'Malley, Rachael Powell, Sharon Hulme, Matthew Lievesley, Wendy Westoby, Jess Zadik, Audrey Bowen, Paul Brocklehurst, Craig J. Smith

**Affiliations:** ^1^ Division of Dentistry School of Medical Sciences Manchester Academic Health Sciences Centre University of Manchester Manchester UK; ^2^ Division of Psychology & Mental Health School of Health Sciences Manchester Academic Health Sciences Centre University of Manchester Manchester UK; ^3^ Division of Cardiovascular Sciences School of Medical Sciences Manchester Academic Health Sciences Centre University of Manchester Manchester UK; ^4^ Salford Royal NHS Foundation Trust Salford UK; ^5^ School of Design Northumbria University Newcastle upon Tyne UK; ^6^ Public Contributor Leigh UK; ^7^ Division of Neuroscience & Experimental Psychology School of Biological Sciences Manchester Academic Health Sciences Centre University of Manchester Manchester UK; ^8^ NWORTH Clinical Trials Unit University of Bangor Bangor UK; ^9^ Division of Cardiovascular Sciences School of Medical Sciences Lydia Becker Institute of Immunology and Inflammation Manchester Centre for Clinical Neurosciences Manchester Academic Health Science Centre University of Manchester Manchester UK

**Keywords:** behaviour, dentist, oral health, oral hygiene, qualitative research, stroke, stroke rehabilitation, toothbrushing

## Abstract

**Background:**

Dental disease is highly prevalent in people with stroke. Stroke survivors regard oral hygiene as an important, yet neglected, area. The aim was to explore experiences of and barriers to oral care, particularly in relation to oral hygiene practice and dental attendance, among stroke survivors in the community.

**Methods:**

This was a qualitative study incorporating a critical realist approach. Interviews were conducted with community‐dwelling stroke survivors requiring assistance with activities of daily living, and focus groups were held with health and care professionals. Interviews and focus groups were recorded and transcribed verbatim. Thematic analysis was conducted.

**Results:**

Twenty‐three stroke survivors were interviewed, and 19 professionals took part in 3 focus groups. Professionals included nurses, speech and language therapists, occupational therapists, dieticians, professional carers and dental staff. Interviews revealed difficulties in carrying out oral hygiene self‐care due to fatigue, forgetfulness and limb function and dexterity problems. Routine was considered important for oral hygiene self‐care and was disrupted by hospitalization resulting from stroke. Professionals highlighted gaps in staff training and confidence in supporting patients with oral care. Access to dental services appeared particularly problematic for those who were not registered with a dentist pre‐stroke.

**Conclusion:**

Oral hygiene routines may be disrupted by stroke, and resulting disabilities may make regular oral self‐care more difficult. This study has identified specific barriers to oral hygiene self‐care and dental service access. Findings from this study are feeding into the development of an intervention to support stroke survivors with oral care.

## 1 INTRODUCTION

Dental disease is highly prevalent among stroke survivors. Untreated tooth decay was found in 48% of patients admitted to a stroke unit in North West England, and 61% had marked gum disease.^1^ This compares to 30% and 50%, respectively, for adults of similar age in the UK general population.^2^ Evidence suggests an inflammatory pathway may link oral health and stroke.^3^, ^4^ Periodontal disease is associated with stroke in epidemiological studies^5^, ^6^ and shares common risk factors such as smoking.^7^ Survivors of stroke have fewer teeth than individuals without prior stroke, and more often wear dentures.^8^ Xerostomia (dry mouth) is a common side‐effect of stroke‐related medication,^9^ and can increase the risk of tooth decay, periodontal disease and oral infection (eg oral thrush) and impact upon wearing dentures. In turn, poor oral health has been linked with important sequelae of stroke, such as aspiration pneumonia,^10^ reduced quality of life and nutritional status.^11^ Tooth decay and periodontal disease can be prevented or effectively managed with appropriate oral health behaviours. Two important behaviours are regular toothbrushing with a fluoridated toothpaste^12^ and visiting a dentist.

Stroke is the leading cause of complex adult disability worldwide, and approximately half of the 1.2 million stroke survivors in the UK are left with disability.^13^ For example, people with stroke may experience complex difficulties due to motor weakness (face, hand/arm), visual field defects, visuospatial problems, aphasia, apraxia and swallowing problems, which can impact on their ability to self‐care and access services. Problems encountered include manual dexterity and coordination difficulties when using a toothbrush, difficulties when rinsing and a diminished awareness when there is food or residue left in the oral cavity.^11^

Approximately one third of patients discharged from hospital after a stroke require help with activities of daily living.^13^ For many people, the impact of their stroke is greatest once they have been discharged home from hospital services, with lack of information provision, access to services and support identified as key factors.^14^, ^15^, ^16^ However, the literature is scant about the oral health needs of individuals discharged from hospital, with most research focussing on oral care in the hospital setting.^17^, ^18^, ^19^, ^20^, ^21^, ^22^ The present study is focussed on stroke survivors discharged to their own homes, or those of family members, in the community. Community‐dwelling individuals must adapt to living independently, with or without support from informal or professional carers. It is important to fully understand the challenges surrounding oral health‐care behaviours for this population to enable appropriate support to be put in place to optimize oral health care and to minimize the negative health consequences of poor oral health care.

This study was in two phases. Phase 1 used qualitative methods to explore experiences of oral care in depth with community‐dwelling stroke survivors and health and care professionals (HCPs) and to identify the specific problems encountered regarding oral health care. The second phase used the findings from phase 1 to inform the development of an intervention to improve oral health among stroke survivors. This paper reports phase 1 only.

## 2 METHODS

### 2.1 Ethical statement

This study was reviewed and granted favourable ethical opinion by NRES Committee Northwest Haydock Research Ethics committee (REC Ref No: 17/NW/0335). The study was conducted according to the standards of the European Medicines Agency Guidelines for Good Clinical Practice. Participants gave informed consent. Confidential information was securely stored.

### 2.2 Design

This was a qualitative study. Semi‐structured interviews were conducted with stroke survivors, and focus groups were conducted with HCPs.

A critical realist approach was taken. Critical realism generally seeks to describe and explain phenomena and people's beliefs, knowledge and understanding of these phenomena.^23^ Physical objects, structures and processes (phenomena) and people's beliefs about these are treated as equally real, connected and thought to be mutual influences upon each other. This approach allowed for the investigation of phenomena, beliefs and the interaction between these. We took the perspective that ‘reality’ has an objective existence that is independent of individuals’ perceptions and understandings, but that an understanding of ‘reality’ can only be accessed through individuals’ interpretations.^24^ During data collection and analysis, we focussed on understanding the perceptions and interpretations of participants from a range of perspectives (stroke survivors and HCPs of various disciplines) in order to gain insights into the issues of importance.

### 2.3 Participants

Stroke survivors were eligible for inclusion if they were aged over 17 years, were diagnosed with stroke (ischaemic or haemorrhagic), had been discharged from hospital for at least 2 months and had difficulty with at least one aspect of self‐care (as assessed by stroke survivors or their carers). Stroke survivors were excluded if they were unable to give consent or lacked adequate understanding of English prior to stroke. Initially, stroke survivors were identified from clinics at an NHS hospital in the North West England. Eligible stroke survivors were mailed study details and invited to contact the study team if interested. No incentive was offered for participation. This method yielded insufficient uptake, and contingency plans were actioned. A researcher attended local stroke survivor support meetings, explained the research and collected contact details of interested individuals. Recruitment to the study occurred between November 2017 and November 2018. Recruitment was guided by the principle of data saturation: data were collected until no new themes arose.

HCPs were eligible to take part if they had experience of working with stroke survivors. Relevant professions were considered to be but not limited to: nurses, occupational therapists, physiotherapists, speech and language therapists, dental professionals, professional carers and dieticians. Individuals were identified through clinical service managers based in the NHS hospital trust, community service contacts in the local area, and email and poster advertisement.

### 2.4 Data collection

Stroke survivors took part in interviews with the researcher individually. Most interviews took place in participants’ homes; six were conducted in public spaces (church halls, community meeting spaces). Topics discussed in interviews included knowledge and understanding of oral care and experiences and perceptions of oral hygiene self‐care and dental visits post‐stroke. The interview schedule included open questions based on the COM‐B model: a model that recognizes that ‘Capability’, ‘Opportunity’ and ‘Motivation’ are important for understanding ‘Behaviour’.^25^, ^26^ An assessment of disability post‐stroke, the modified Rankin Scale,^27^ was carried out to describe the sample. This scale assesses disability in a person who has had a stroke, and scores range from 0 (no symptoms) to 5 (severe disability, bedridden, incontinent, requiring constant nursing care). Stroke survivors were asked to report their age bracket, gender and whether or not they lived alone. Interviews ranged from 35 to 60 minutes; some were cut short due to participant fatigue. Family member carers supported participants with aphasia or cognitive deficits as required. Interviews were audio‐recorded and transcribed verbatim.

The focus groups with HCPs were conducted on hospital premises between November 2017 and May 2018. Topics discussed included the importance of oral care, experiences in supporting patients with oral care and what might be most helpful for improving oral self‐care for stroke survivors. Each focus group included HCPs from more than one discipline. This allowed ideas and issues to be discussed from different perspectives, and issues to be raised and considered in a way that may not have occurred without these contrasting viewpoints. The focusgroup facilitators took care to ensure that participants of different backgrounds and perspectives were able to voice their thoughts and views. Focus groups were audio‐recorded and transcribed verbatim. Focus groups ranged in length from 70 to 95 minutes.

### 2.5 Analysis

A thematic analysis^28^ was conducted, supported within NVivo 12. The analysis began with familiarization with the data, which was achieved through reviewing the transcripts. Initial codes were generated and sorted into themes. This was an iterative process between two members of the research team. Following this, the themes were reviewed, refined and named.

Data from the interviews and focus groups were analysed together. Analysis was carried out concurrently with data collection so that data saturation could be assessed. Data saturation was assessed and agreed upon by three members of the research team on an on‐going basis.

## 3 RESULTS

Interviews were conducted with 23 stroke survivors. They ranged in age from 28 to 94 years, nine were female, and all lived in the Greater Manchester area. Ten lived alone (three lived alone in sheltered accommodation); thirteen lived with a partner. The mean modified Rankin Scale score was 2.4 (range 1‐4).

Nineteen HCPs participated in three focus groups. Focus group 1 consisted of two dieticians and seven speech and language therapists; group 2 contained two nurses, two occupational therapists and one physiotherapist; group 3 consisted of two dental care practitioners, one consultant in restorative dentistry, and three professional carers.

The findings are presented as they relate to two dental health behaviours: (a) oral hygiene and self‐care behaviour and (b) dental attendance.

### 3.1 Oral hygiene and self‐care behaviour

All stroke survivors reported that they took part in oral hygiene activities (toothbrushing or mouth cleaning) to some extent, and HCPs recognized toothbrushing or mouth cleaning as something that was important for their patients. The following themes are presented: knowledge and beliefs, routine, disability as a result of stroke and support and training.

#### 3.1.1 Knowledge and beliefs

Participants’ beliefs around what oral care behaviours they thought they should carry out, and thoughts about why oral care behaviours were important, were discussed. The analysis of perceptions around knowledge and beliefs focussed on the perspectives of stroke survivors, rather than on those of the HCPs who were likely to be taking part with professional interest and expertise in oral care. Stroke survivors generally reported an understanding of what they should do that was consistent with guidelines, for example,

Clean them. Just keep yourself healthy. Like I say, brush regularly. Not too many sweeties. Can’t think of anything else (Stroke Survivor (SS)10)

Others seemed to have a less clear understanding. For example, SS11 appeared to be keen to follow dentist advice: they did not rinse their mouth with water after cleaning their teeth:

And then I finish brushing my teeth then I use mouthwash… Because that toothpaste, the dentist told me not to rinse it out [with water] (SS11)

However, rinsing with mouthwash after brushing has the same negative impact as rinsing with water and is also not recommended. This illustrates a general desire of participants to look after oral hygiene, and motivation to carry out recommended behaviours, but it appears that a lack of knowledge or understanding of oral care guidance could lead to suboptimal self‐care.

When discussing why oral hygiene was important, few stroke survivors appeared to consider a connection between oral hygiene and general health. It is unclear whether this was because participants were unaware of the connection, or whether such a connection was not salient to them, particularly in the context of wider health issues resulting from the stroke. However, it seemed that oral hygiene was important to participants from an aesthetic perspective: the desire to look and smell good:

And I was quite concerned that I wasn’t properly doing it. Kept saying to her, if I’m smelly tell me, tell me, you know, what…and she said, well your breath’s not so good. And that then got me (SS11)

Avoiding smelly breath was clearly critical to this individual, and learning of smelly breath seemed to cause upset and shame. Social presentation, and its impact on social interactions, seemed of high importance to participants, and so oral care appeared to be highly valued.

This theme illustrates that participants strongly believed oral care to be important, with its value relating, for some, to social presentation. Some stroke survivors appeared to lack knowledge of specific recommended oral care behaviours, and may lack awareness of general health benefits of keeping the mouth clean (rather than specifically dental health benefits). Understanding stroke survivors’ knowledge and beliefs about oral care is important as if people lack an understanding of what to do, or do not feel a behaviour to be important, they may be less likely to enact that behaviour.

#### 3.1.2 Routine

Routine appeared to be a key factor for many of the stroke survivors in determining whether they brushed their teeth regularly. Some stroke survivors described current successful routines that ensured they did not forget to brush, for example,

Because I do, just to freshen my mouth when I get up in the morning. Because I'm doing the shower in the morning, it gets to be a routine. I forget at night. (SS19)

For this participant, where brushing was embedded in their morning self‐care routine, it was regularly conducted. However, at night‐time, where it was not part of such a routine, there was a tendency to forget.

Stroke survivors spoke about how changes in circumstances caused by stroke could lead to disruptions in routines. In particular, being hospitalized post‐stroke could severely impact oral care routines, through taking a person out of the environment in which a routine was embedded, and their being too unwell to look after their own oral care:

when you’re in that kind of state, and you’re not feeling yourself, and you’re out of home, you’re not in your routine, so you don’t really think about, oh well, I should brush my teeth now… So, I did find it, in hospital, probably I hardly brushed my teeth really, at all, because quite often I didn’t want to ask, or couldn’t be bothered, or was too tired, or whatever, like, when you can’t do it yourself, it just feels like you don’t want to do anything. (SS4)

When I went into hospital I didn’t brush them at all in the hospital and I was in there for seven months. So when I come out it was easy, the same, carrying on doing the same … I was brushing my teeth every day up till then and then in hospital I didn’t bother, and it’s so hard to get back into (SS16)

This second quote illustrates how having a habit broken during hospitalization could impact a person's routine after discharge. These quotes suggest that stroke survivors may be receiving inconsistent or absent support for oral care in hospital, and this could potentially impact patients’ oral care routine in the longer term. To fully understand barriers to oral care after discharge to the community, it seems to be important to also understand what happens with oral care during hospitalization such that teeth cleaning may not regularly occur.

Discussions in focus groups with HCPs confirmed a sense that oral care could be neglected during hospitalization:

Because even in hospital, there's care plans for all sorts of things, but there isn't an oral hygiene care plan, is there? (FG1)

A speech and language therapist said:

That probably gives you an idea of how often people don't look in people's mouths, that you might be the first person to identify thrush, when they've been in hospital for a little while. And it's pretty evident when you look at it that they've got a tongue that's covered and looks like they've had thrush… But it does make you think how has no one seen that? (FG1)

One reason for this neglect in hospital seems to be a lack of clarity as to whose responsibility it is to ensure that oral health care is provided.

there aren't any written leaflets for mouth care, are there, that we could give out. And I think it probably again comes down to that whose responsibility … who should do that? (FG1)

But it's the nurse's job … well they do washing and dressing, don't they, and how many times do we get, we've done everything but we've not done their teeth (FG2)

Hospitals were recognized as busy workplaces with many competing demands that may lead to oral care being overlooked. One speech and language therapist stated,

There's so much responsibility on nurses to cover everything. [Oral care] just feels like it is this extra luxury that might be missed. But sometimes you do go to a staff member and say can you just come and look at this person's mouth?… And when you know someone's really pressured or you're up on critical care and you think there are different pressures, people are in very, very medically unwell situations. You almost feel like… I'm just showing you this and I know it's not a big deal, but it actually is a big deal (FG1)

This quote reflects the context of stroke survivors’ care during hospitalization. Focus‐group participants recognized that, during the hospitalization period, stroke survivors may be very unwell, and may have complex care needs and comorbidities. The ward staff were seen as being very busy in looking after these unwell individuals, such that oral care was not always prioritized. Focusgroup members understood these challenges but also seemed to feel that oral care should receive more attention.

Thus, routine seemed to be important in helping stroke survivors to maintain their oral care behaviours, but hospitalization could disrupt routine and oral care long‐term. It would appear that there are complex, systemic reasons for a lack of support for oral care during hospitalization.

#### 3.1.3 Disability resulting from stroke

Disabilities caused by the stroke appeared to cause problems for oral care. Although the ability to perform self‐care tasks such as teeth cleaning improved over time for some, others had continuing disabilities that impeded their abilities to care for their own mouths. Some participants had physical impairments, which made teeth cleaning challenging:

I lost the use of my dominant hand, cleaning your teeth when you can’t open your mouth and it’s the wrong hand it’s difficult (SS12)

A particular problem raised by a number of participants was difficulties in putting toothpaste on the toothbrush, for example:

I have to wedge my toothbrush, you know, in‐between the tap and the wall, and that, and put the toothpaste on. That's the hardest bit. (SS7)

The toothpaste, the actual toothpaste, I push up against the nearest part of the sink to me so I unscrew and put the toothbrush wedged in the draw so I can squeeze it out then (SS11)

A clear problem therefore is reduction in dexterity such that survivors had the challenge of conducting a two‐handed task with only one hand, were learning to clean their teeth with the non‐dominant hand, or were experiencing difficulties whilst still using the impaired dominant hand. Nevertheless, the dexterity problems did not deter people from toothbrushing, and some creative solutions were described for challenging processes such as putting paste on the brush, as indicated in the above quotes.

One participant described mobility difficulties that made it difficult for her to move to the bathroom to brush her teeth; this left her reliant on support from family members or carers to bring the equipment to her:

normally, if I had to brush my teeth and I couldn’t use the bathroom upstairs I would use that sink in the extension, but I wouldn’t be able to get out there because even with an electric wheelchair I wouldn’t be able to get down the steps (SS14)

Stroke survivors mentioned impacts of stroke on cognitive processes, such as memory, as well as physical limitations related to toothbrushing. Fatigue and forgetfulness seemed to disrupt toothbrushing:

I always clean my teeth at least once a day, sometimes I’m tired and I can’t be bothered, I’ll be honest with you (SS6)

[why don’t you brush your teeth at night?] ‘Because I forget. I do sometimes but I forget … I don't know, just laziness as well. Because I have my shower and everything in the morning. If I had a shower at night, I'd brush my teeth as a routine (SS19)

The use of the term ‘laziness’ by some of the participants would seem to suggest that they took responsibility for cleaning their teeth, and blamed themselves for times when they did not manage to do this. However, participants who used this term did seem to perceive oral care to be important, and it seemed that issues such as fatigue seemed to influence whether or not teeth cleaning was actually carried out. As such, stroke survivors may be putting themselves down for neglecting their oral hygiene when it may be more a consequence of stroke‐induced fatigue.

HCPs spoke of the difficulties they had witnessed stroke survivors encounter when brushing their teeth. Some of their observations were similar to issues raised by stroke survivors, for example problems caused by mobility and dexterity limitations:

So they might be able to use their arm to brush their teeth, but everything is left completely out of reach for them (FG1)

the difficulty I had with somebody, was getting the toothpaste out of the tube…so what they were doing was having to buy new toothpaste all the time. Because it got to a certain level, but they couldn't squeeze it up anymore (FG2)

HCPs identified some cognitive and sensory issues that were not raised by stroke survivors:

I think sometimes, well in some cases, it's cognition, so not being able to recognise that they need to do it as part of a routine (FG2)

toothpaste is often a difficulty, because a lot of them are white. So you have a white toothpaste, in a white bathroom, in a white cup. So someone with visual difficulties who needs contrast, can barely see that, to be able to take the lid off, to be able to use a toothbrush (FG2)

It is not clear why such cognitive problems were not also raised in stroke survivors’ interviews. It may be that the individuals who were willing to take part in interviews were those who were less likely to be experiencing these particular cognitive difficulties, or it may be that such problems were experienced but not recognized by some stroke survivors. Overall, a wide range of physical and cognitive disabilities seemed to impact stroke survivors’ abilities to clean their teeth, and the exact difficulties experienced by individuals could vary.

#### 3.1.4 Support and training carers

A number of stroke survivors reported that they relied on support from family members and HCPs.

I did have help, because I didn’t come straight here, I moved in with my mum and dad, for about four or five weeks or so, after I came out of hospital. So, they helped me to…not to brush, but I’d need someone to at least put the toothpaste on the brush for me, and then just give me the brush (SS4)

[wife’s name] said, ‘you’ve got to clean your teeth this morning’… And then that’s a reminder, go and clean your teeth. And usually I do remember, but sometimes I don’t, and my back‐up is [wife’s name] (SS13)

It seems that the support welcomed by stroke survivors was in line with the difficulties they personally experienced after the stroke: helping with the physical act of putting the paste on the brush for someone with physical impairments, and reminding a survivor with a tendency to forget. One participant described a feedback game that he and his wife had designed to encourage him to do his morning washing and dressing routine successfully.

I brush my teeth… I go back in the bedroom and aim for a medal… Because my sister phones every day…and she said, ‘what medal did you get today’, because we have a laugh about me getting a bronze, a gold, silver or brass, and [my wife] gives me an award every day for how I shape myself (SS8)

In the above examples, stroke survivors were able to manage self‐care with support from carers. Other stroke survivors required higher levels of care. In such instances, appropriate training appeared to be important. The wife of a stroke survivor who had no teeth but required regular mouth care described how being shown how to care for her husband by hospital nursing staff was critical to her being able to help him,

Yeah, we were provided with the Biotene gel and when he was on the ward…they showed me how to…clean his mouth and that while he was on the ward so that I could do it when I was in. So, no, they were really good [had they not have shown me] I won't have really known anything (SS9)

The need for training for HCPs responsible for stroke survivors’ care was raised in focus groups:

The district nurses that he was having in said that it's not for them to clean the patient's mouth because he's nil‐by‐mouth and they don't know what to do. The family are too frightened to do it…And then the carers had said we don't do any of this…putting a toothbrush in somebody's mouth would be invasive, so they wouldn't do it either. And the patient was actually requesting to have his teeth cleaned. And it wasn't done (FG1)

We think there's nervousness amongst some staff now, to really get in there and clean a mouth. Because we work in a really risk averse environment, now, so I think if you're dealing with somebody that's got swallowing issues, and they're nil‐by‐mouth, and there's aspiration risk, I think some staff would take a lot of training to have the confidence (FG2)

These quotes illustrate an apparent lack of confidence around cleaning others’ mouths, which would seem to relate to a lack of skills and training. One particular concern seemed to be the potential for stroke survivors with swallowing problems to inhale substances, such as toothpaste, placed in the mouth. This worry could lead to the HCP avoiding this important element of care. There also appeared to be the problem that, for those working in the community (eg district nurses and carers), it may not be clear whose role it is to look after oral care.

Lack of training was also perceived to cause a lack of awareness of the need for oral care, as one professional carer described:

it’s a little bit forgotten…mouth hygiene…in community, because the carers are not trained. And they are not aware what can cause the problem if you don’t brush your teeth. So, I think…just to give the training to the carers…because you are the carer, and visiting people, so you have a relationship with them, and you start to talk with them, it will be good to encourage them to brush their teeth (FG3)

This participant recognized that not only might a carer be able to support a stroke survivor by physically helping them with oral self‐care, but they could also support oral self‐care by encouraging someone to clean their own teeth.

A key problem limiting professional carers in providing oral care seemed to be that carers can be restricted by what is in an individual's support plan.

C1: I know it sounds stupid, but it’s got to be put in a support plan… Everything, even down to taking rubbish out, has to be put in a support plan, or the carers don’t do it… Social services go out first, and then we get a support plan to detail…

D: And is one of the questions on that, not about oral care?

C1: Yeah but it’s got to be put in the support plan, for us to do it.

C2: One of the questions is, does the service user require assistance with dental hygiene?

D: And so often is that not filled in?

C1: No, it’s usually, no (Discussion between professional carers (C) and a dentist (D) in FG3)

Thus, there seem to be three factors that affect the support carers are able to provide: a lack of training influencing skills, confidence and awareness of oral hygiene issues; lack of clarity around which HCP’s role it is to look after oral hygiene; and a failure in systems such that the need for oral care can be missed from support plans.

### 3.2 Dental attendance

With regard to dental attendance, one of the biggest challenges for participants seemed to be perceived access to services. There were two aspects to this. Firstly, disabilities from stroke physically restricted access,

He's moved and it's upstairs now and there's no lift. So, it is difficult because they've only got a rail on one side and anyone disabled now wouldn't be able to get up there. (SS19)

Secondly, some participants could not find a dentist willing to take them on. Generally, those registered with a dentist prior to stroke seemed better able to access care post‐stroke; those who did not have a regular dentist experienced difficulties,

we don't have a regular dentist and I spoke to a local one and she advised we…ask either the GP or one of the therapists to refer him to the community one [dentist] for the ones that deal with people with disabilities. But I mean that's been a while since, I know [speech therapist] sent a referral off, but we've not heard anything (SS9)

One of the dental health practitioners who worked in the community dental service referred to what she felt were inappropriate referrals from primary care,

It’s becoming such a strain on community dental services as well…I think someone that’s recovered from a stroke, and has mild problems, should still be able to be managed in a general dental practice setting, like not much of their life should change really, it should have to have a few adaptations, but, they’re just getting referred (FG3)

Thus, it would seem that from a dental care perspective there is no reason why general dental practices should not be able to provide care for many stroke survivors, but barriers appear to remain for dental practices when taking on stroke survivors as new patients. There is a need to ensure that stroke survivors are able to physically access dental practices, and to understand and address the systemic issues that appear to be restricting access to dental care in a primary health‐care setting.

## 4 DISCUSSION

This study's findings revealed that oral self‐care was highly valued—in particular in terms of self‐presentation—but that some stroke survivors lacked full awareness of oral care behaviour guidance. Difficulties in enacting oral self‐care arose from physical and cognitive disabilities resulting from stroke. Furthermore, oral self‐care was inhibited by disruption to routine during hospitalization. Carers had important roles in supporting the oral care of survivors, but lack of training and systematic problems could be barriers to providing such support. There were physical and systemic issues that led to difficulties in accessing primary care dental services.

This study has highlighted a particular challenge around oral care and stroke in that the problems are very individual in nature. There is substantial variation in the type and severity of disability that survivors are left with following their stroke. These variations create a multitude of different ways in which survivors may need to be supported in looking after their oral health.

It is striking that oral care post‐stroke seemed to be negatively impacted by hospital stays where patients were not supported in looking after their mouths. Hospital stays may represent important moments for intervention in oral health either to maintain self‐care or to teach and support those who do not regularly clean their mouths. This is particularly important considering the links between complications of stroke and poor oral hygiene.^29^, ^30^ However, it is apparent that there are a number of factors that mean oral care can be neglected in hospitals such as limited staff and resources, lack of training and confidence, lack of role clarity and a lack of awareness around oral care. To further compound the problem of losing their routine in hospital, stroke survivors may return to their homes unable to easily reengage with their oral care routine due to disability.

There have been few studies that have looked at the experiences of stroke survivors regarding their oral health. A mixed‐methods study^17^ that investigated oral care in stroke units through interviews with patients found that oral care was a neglected area of care and that there was a lack of awareness about oral care and a lack of available advice and information. Although the focus of the study by Horne and colleagues^17^ was during hospitalization and our study's focus was post‐discharge, there is concordance in findings, particularly regarding participants’ experiences in hospital. Furthermore, focus groups with health professionals in Horne et al’s study highlighted similar issues around the lack of training for staff around supporting patients with oral care.

With regard to the importance stroke survivors place on their oral health, our study found that many people seemed most concerned about their appearance and the social consequences of poor oral health such as bad breath. This fits in well with previous research conducted around Oral Health–related Quality of Life in which the social importance of oral hygiene among stroke survivors has been emphasized.^31^

The strengths of this study lie in its in‐depth exploration of the experiences of stroke survivors, which has elucidated key problems to be targeted by future interventions. This study is limited in generalizability in that all data were collected in one region of England, and this may be reflected in some of the issues that were emphasized particularly around accessing dental services. However, many of the difficulties resulting from varying disabilities and loss of routine due to the disruption to life from stroke are likely to be representative of stroke survivors more widely. The study sample is subject to self‐selection bias in that all participants had to actively volunteer to take part. This may help to explain why the majority of the participants in this study had quite low levels of disabilities. Additionally, exclusion criteria meant that non‐English speakers and those lacking capacity to provide consent were not represented.

A further limitation of this study was that we did not comprehensively investigate the ways in which diet was affected by stroke. The pattern of sugar consumption is a key oral health behaviour because dietary sugars have a direct role in the cause of oral diseases.^32^ Dietary behaviours were raised in interviews, but there was not time to fully explore them without putting too great a burden on participants. We did glean that variation in diets and individual health needs were complex and there may be discordance between traditional dental health advice (restrict all dietary sugars to mealtimes) and advice given out post‐stroke (eat little and often for those who have low body weight). This is a complex area that warrants further investigation.

This study has implications for practice in terms of raising awareness of the need for more support for oral care for stroke survivors. Approaches are needed to enhance stroke survivors’ skills to manage oral care despite physical disabilities, to help them to remember to clean their teeth, and to make sure they understand current oral care guidance. Support is needed for carers (professional carers and family members) to ensure that they have the awareness, skills and confidence to support stroke survivors with oral care. There are also a range of systemic problems that need to be tackled: oral care routine during hospitalization needs to be maintained, not disrupted; clarity is needed as to roles of different HCPs in supporting stroke survivors with oral care; oral care needs to be consistently addressed in support plans; and access to primary dental care needs to be assured.

The findings of this study have fed into phase 2 of this project, where a novel approach combining the Behaviour Change Wheel^25^, ^26^ and evidence‐based co‐design^33^ is being followed to develop interventions to support oral care in stroke survivors. The outcome of phase 2 will be a toolkit co‐designed by patients, carers and professionals, utilizing behaviour change theory, to support good oral care behaviours.

## 5 CONCLUSIONS

It is clear that stroke can have adverse effects on oral hygiene both through the disruption to routines and from the resulting disabilities that survivors are left with. Support however is lacking and could be improved. Following on from this study, a toolkit is being developed to improve support for oral care among stroke survivors.

## CONFLICT OF INTEREST

The authors have no conflicts of interest to declare.

## Data Availability

Research data are not shared. Due to participant confidentiality, we are unable to share the full transcripts of interviews and focus groups collected as part of this study.
